# Factors Associated With Hypertension Awareness, Treatment, and Control Among Adults in Kerala, India

**DOI:** 10.3389/fpubh.2021.753070

**Published:** 2021-11-01

**Authors:** Yingting Cao, Thirunavukkarasu Sathish, Tilahun Haregu, Yu Wen, Gabrielli Thais de Mello, Nitin Kapoor, Brian Oldenburg

**Affiliations:** ^1^Melbourne School of Population and Global Health, University of Melbourne, Carlton, VIC, Australia; ^2^Implementation Science Lab, Baker Heart and Diabetes Institute, Melbourne, VIC, Australia; ^3^Population Health Research Institute (PHRI), McMaster University, Hamilton, ON, Canada; ^4^Research Centre for Physical Activity and Health (NuPAF), Federal University of Santa Catarina, Florianópolis, Brazil; ^5^Department of Endocrinology, Diabetes and Metabolism, Christian Medical College, Vellore, India; ^6^School of Psychology and Public Health, LaTrobe University, Melbourne, VIC, Australia

**Keywords:** hypertension, awareness, treatment, control, risk factors, longitudinal, India

## Abstract

**Background:** Hypertension, the most significant risk factor for cardiovascular disease, is an increasing contributor to global health burden, particularly in low- and middle-income countries (LMICs) such as India. While the rates of hypertension awareness, treatment, and control in India have been reported in several studies, the factors associated with these rates are less well-understood. Existing studies are predominantly cross-sectional, and the factors examined are limited. Understanding the predictors associated with these rates, using more rigorous study designs, is crucial for the development of strategies to improve hypertension management.

**Aims:** To examine a range of factors associated with hypertension awareness, treatment, and control using both cross-sectional and longitudinal analyses.

**Methods:** Data was derived from a population-based sample of 1,710 participants from Kerala, aged 30–60 years. We examined a comprehensive range of factors, including demographic, behavioral factors, anthropometric, clinical measures, psychosocial factors and healthcare utilization. Multilevel mixed effects logistic regression was used for both cross-sectional and longitudinal analyses (repeated measures for all variables across 2 years) to determine the factors associated with awareness, treatment, and control of hypertension.

**Results:** A total of 467 (27.3%) participants had hypertension at baseline. Among those, the rates of awareness, treatment, and control of hypertension were 54.4, 25.5, and 36.4%, respectively. Being male (OR 0.27, 95% CI 0.14–0.53) and consumption of alcohol (OR 0.49, 95% CI 0.31–0.80) were significant predictors of poorly controlled hypertension (longitudinal analysis). Depression (OR 2.04, 95% CI 1.15–3.61) and fair-to-poor self-perceived health status (OR 1.87, 95% CI 1.15–3.04) were associated with increased hypertension awareness, whereas anxiety (OR 1.97, 95% CI 1.04–3.71) was associated with increased hypertension treatment (cross-sectional analysis). Seeking outpatient service in the past 4 weeks was associated with higher awareness (OR 1.09, 95% CI 1.27–2.87), treatment (OR 1.73, 95% CI 1.20–2.50) and control (OR 1.96, 95% CI 1.37–2.80) (longitudinal analysis).

**Conclusion:** Our findings suggest the importance of considering psychosocial factors and better engagement with health services in hypertension management, as well as giving more attention to body fat control and largely male-related behaviors such as alcohol consumption, taking into account of some Indian specific attributes.

## Introduction

Hypertension remains one of the most important modifiable risk factors for the morbidity and mortality associated with cardiovascular disease (1). While hypertension control has improved over the past few decades globally, the prevalence has increased in low- and middle-income countries (LMICs), with low proportions of hypertension awareness, treatment, and control (2). In India, there has been a big increase in the prevalence of hypertension over the past two decades (23–42.2% in urban and 11.2–28.9% in rural areas), with no substantial improvement in the rates of hypertension awareness, treatment, and control (3).

Improved awareness and treatment of hypertension can lead to improved control of hypertension (4, 5). Understanding which factors are associated with these rates is very important for developing appropriate strategies to improve hypertension control. Studies conducted in LMICs have investigated demographic and behavioral factors that can be associated with hypertension awareness, treatment, and control. Some of these studies suggest that, being a female, overweight or obese, non-smoker and non-drinker are associated with higher rates of hypertension awareness and/or treatment or control (6, 7). Others have found higher waist-to-height ratio and having co-morbidities (e.g., diabetes and other chronic conditions) are associated with higher rates of hypertension awareness or treatment (8); physical inactivity was associated with higher levels of treatment, and higher percent of body fat was associated with higher level of awareness, treatment, and control (9). However, all these studies were cross-sectional; further prospective studies are needed to determine the predictors of these important rates in hypertension management. In addition, investigating other factors in addition to demographic and behavioral factors in relation to hypertension awareness, treatment, and control may also be important to help manage hypertension (10).

There are also psychosocial factors such as anxiety and depression that have been shown to be associated with the development/worsening of hypertension (11, 12), yet, they haven't been extensively explored in relation to awareness, treatment, and control of hypertension. Similarly, health-related quality of life has been shown to be poor in patients with hypertension (13), but it is not clear how it can be important in relation to hypertension awareness, treatment, and control. Social support, including family and friends' ties, was shown to be associated with less uncontrolled hypertension (10). On the other hand, health service utilization factors, including easy access to health services and regular check of BP, were both found to be associated with higher hypertension awareness (9). Taken together, more scrutiny is needed to further examine the role of a wider range of factors that includes not only demographic and behavioral factors, but also psychosocial factors in relation to hypertension awareness, treatment, and control.

In India, several studies have investigated the prevalence of hypertension awareness, treatment, and control (14–16). Factors associated with these rates were only explored in cross-sectional studies and also, only a limited number of potential factors were investigated (9). Considering all potential factors from different aspects mentioned above in relation to hypertension awareness, treatment, and control, not only in cross-sectional, but also in longitudinal studies, could enhance our understanding of the determinants of these rates and how to improve hypertension control in the population. This will help establish stronger evidence-based strategies to reduce the burden of cardiovascular diseases in India and other LMICs.

Our team has established a cohort in Kerala India in 2013, to implement and evaluate a community-based diabetes prevention [Kerala Diabetes Prevention Program (K-DPP)] (17). Despite being an intervention trial, comprehensive information was collected on all study participants, including socio-demographic measures, behavioral measures, psychosocial measures, clinical and biochemical measures, as well as measures of health utilization. This has enabled us to examine a range of factors that could be associated with hypertension awareness, treatment, and control in a population representative sample. Moreover, three repeated measures at baseline, 12 and 24-months means that it is possible to examine such associations longitudinally in order to increase the understanding of the casual relationships between a diverse range of factors and hypertension awareness, treatment, and control.

Therefore, the current study aimed to examine a range of predictors of hypertension awareness, treatment, and control, using both cross-sectional and longitudinal analyses in the cohort of K-DPP. The predictors to examine include demographic variables, behavioral factors, anthropometric and clinical measures, psychosocial factors (i.e., anxiety, depression, chronic stress, social support, and health-related life quality) and measures of health utilization.

## Materials and Methods

### Study Participants

**Figure 1:**
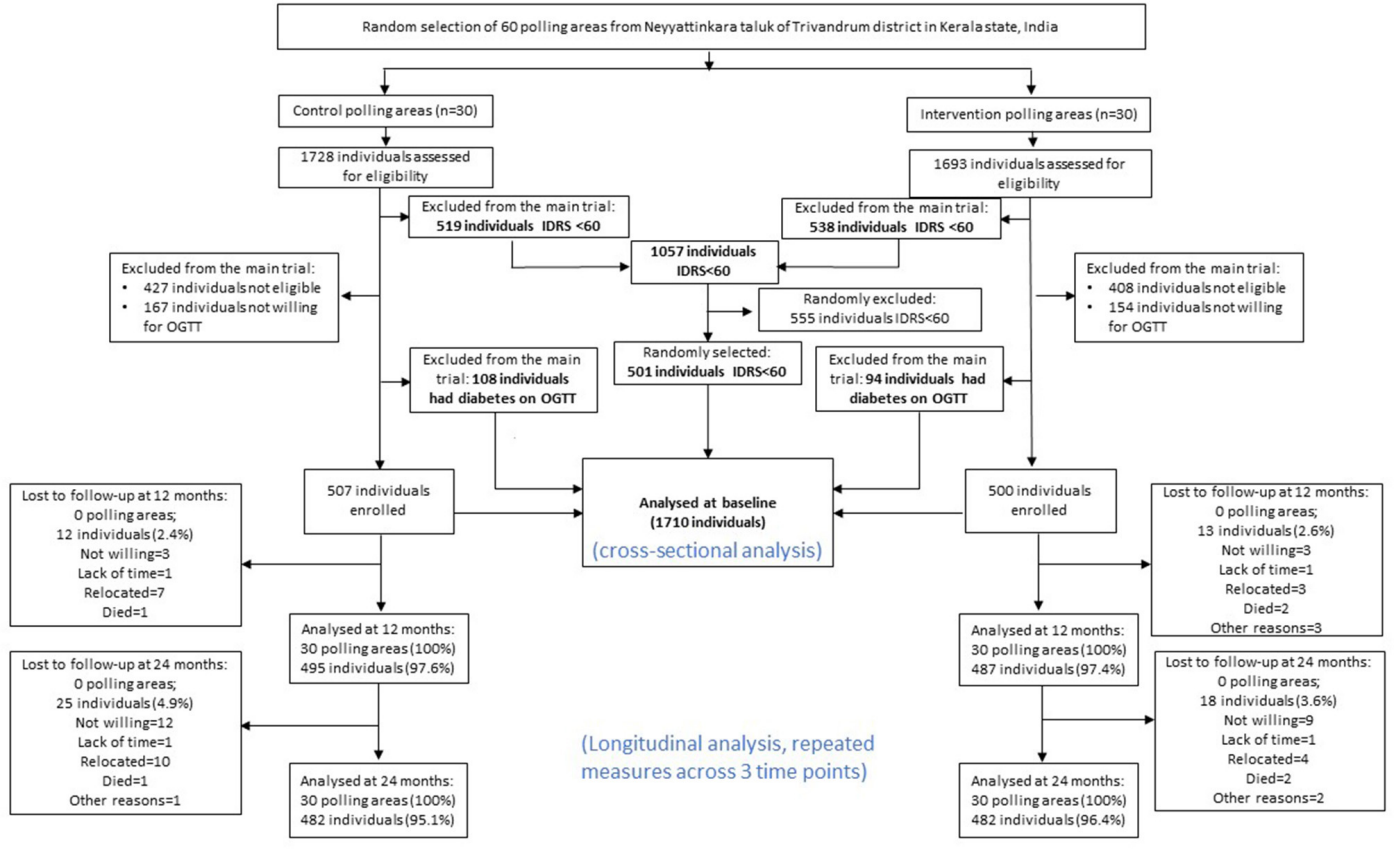
Flow chart of the present study, adapted from previous K-DDP publications (20). IDRS, Indian Diabetes Risk Score; OGTT, oral glucose tolerance test.

Study participants in this study were a broadly representative, population-based sample from the general population in Kerala, aged from 30 to 60 years, collected in 2013 (18). The detailed information on study design and participants screening and recruitment have been previously published (17, 19). Detailed recruitment information is presented in Figure 1.

After first step of excluding IDRS <60 (*n* = 1,057) and those who were not willing to have OGTT (*n* = 320), 1,209 participants underwent the OGTT to assess the presence of diabetes. A total of 1,007 participants who were free of diabetes at baseline were included in the K-DPP trial (intervention group 500, control group 507). For those who with IDRS <60 (*n* = 1,057), a random selection of participants (*n* = 501) were recruited. All measures for these 501 participants were done as were for the trial population. For the purpose of this study, to include more general population, we included K-DPP trial participants (*n* = 1,007), those who had diabetes at baseline (*n* = 202), and those who with IDRS <60 at baseline (randomly selected *n* = 501), resulting in a total study sample of 1,710 at baseline for analysis (Figure 1).

### Data Collection and Measurements

A range of variables were measured including socio-demographic measures, behavior measures, clinical measures, psychosocial measure and cost-effective measures, which have been published previously (17). Data collection was performed by trained staff, using standard questionnaire, in accordance with WHO STEPS protocol (21). Anthropometric measures were performed using standard protocols (22), including height, weight, waist and hip circumferences, and body composition. Blood pressure (BP) was recorded three times using the Omron automatic BP monitor (model IA2) with an interval of at least 3 min between the readings. BP monitors are calibrated weekly using a sphygmomanometer. The average of the second and third BP readings was used in the current analysis. Standard protocols were followed for collection of fasting glucose, OGTT, HbA1c, and lipids (17). Blood samples are centrifuged within 30 min at the clinic and transported to a laboratory accredited by the National Accreditation Board for Laboratories (NABL) (23) for processing. Family history of CVD was also assessed by the question: “Do you have any family history of cardiovascular disease (heart disease or stroke)?”.

We defined hypertension as mean systolic BP (average of second and third measures) ≥ 140 mmHg or diastolic BP ≥90 mmHg based on the JNC 8 classification of hypertension (24), or currently under anti-hypertensive medication or self-reported hypertension. In order to better understand the stages of hypertension in this sample, we have further checked prehypertension (systolic BP between 120 and 139 mmHg or diastolic BP between 80 and 89 mmHg); stage 1 hypertension (systolic BP between 140 and 159 mmHg, or diastolic BP between 90 and 99); stage 2 hypertension (systolic BP between 160 and 179 mmHg, or diastolic BP between 100 and 109); and stage 3 hypertension (systolic BP ≥ 180 mmHg, or diastolic BP ≥ 110 mmHg) (24). We defined hypertension awareness as self-reported previous diagnosis of hypertension by a physician among those with hypertension. Similarly, we defined hypertension treatment as currently taking anti-hypertension medication. Controlled hypertension was defined as systolic BP <140 mmHg and diastolic BP <90 mmHg among those hypertensive participants (regardless of receiving treatment) based on the definition mentioned above.

### Behavioral Factors

Tobacco use was assessed by asking the question “Did you use any of the following tobacco products (smoking: cigarettes, bidis, cigars and hookah; smokeless: snuff, betel with tobacco, khaini and gutka) in the last 30 days?” Fruits and vegetable intake were assessed using Food Frequency Questionnaire adapted from PROLIFE study (25). Alcohol consumption was assessed by asking the question “Did you consume an alcoholic drink (such as beer, wine, whiskey, toddy) in the last 30 days?” Self-reported levels of physical activity were measured using the Global Physical Activity Questionnaire (26).

### Psychosocial Factors

Anxiety was measured using the General Anxiety Disorder scale (27). Depression was measured using Patient Health Questionnaire-9 amended in line with CURES-65 study (28). Chronic stress was measured by Chronic stress scale used in MESA study (29). Self-perceived health status was measured using Short Form-36 (30).

Social support was measured by the ENRICHED social support scale (31), consisting of 7 items with 5 scale (total score = 35), with higher score indicating higher level of social support. Health-related quality of life was assessed using the 36-item Short-Form (SF-36) health survey (30).

### Health Utilization

Health utilization was assessed by asking the questions “Did you have any out-patient services during the past 4 weeks (including specialist, community health services, nurses etc.) and “Did you have any in-patient services in the last 1 year?”

### Statistical Analysis

Descriptive statistics were used to present participant characteristics in general and by hypertension awareness, treatment, and control. Mean (SD) and proportions (%) were used to summarize for continuous and categorical variables, respectively. Chi-square test was used to compare difference between proportions, and ANOVA was used to compare differences in continuous variables between groups. Multilevel mixed logistic regression (25) was performed for both cross-sectional and longitudinal analyses, considering polling areas (clusters) as the second level in the model. Odds ratios were obtained by exponentiating the estimated regression coefficients were presented. For the longitudinal analysis, repeated measures for all variables (predictors and outcome variables) at all three time points (waves) among trial participants detected with hypertension were included in the model, adjusting for wave and study arm. Variables tested in the univariate analysis include demographic factors, behavioral factors, anthropometric and clinical measures (including the family history of CVD), and health utilization. Variables selected in the final multivariable analysis for each of the outcome variables was decided based on evidence from previous studies and significant results of univariate analysis. All analyses were conducted using STATA16.0 (College Station, TX: StataCorp LLC).

## Results

In total, there were 1,710 participants included in this study at baseline (62% male, mean age 45 years, SD 7.9 years). The prevalence of hypertension was 27.3% (*n* = 467, including 170 with controlled hypertension) at baseline. Characteristics of participants with hypertension at baseline (*n* = 467) were presented in Table 1. The prevalence of hypertension awareness, treatment, and control is presented in Table 2. In brief, among all hypertensive participants (*n* = 467), 54.4% were aware of their hypertension [indicating 45.6% (213 participants) were newly diagnosed with hypertension], 25.5% were receiving treatment and 36.4% had their BP controlled at baseline. Among those who were aware of their hypertension, about 47% were receiving treatment, and among those with treatment, about 69% had their BP controlled. Among those with uncontrolled hypertension (*n* = 297), 72.5, 20.9, and 7.1% had stage 1, stage 2, and stage 3 hypertension, respectively.

**Table 1:** Characteristics of participants with hypertension at baseline (*n* = 467).

**Factors**	***n* (%)**
***Demographic factors***	
Age (years), mean (SD)	47.6 (8.0)
Men	308 (66.0)
Marital status, *n* (%)	
Married	451 (96.6)
Not married (separated/ divorced/widowed/never married)	16 (3.4)
Education, *n* (%)	
Up to primary school	142 (30.4)
Secondary school	263 (56.3)
Tertiary and above	62 (13.3)
Occupation, *n* (%)	
Skilled/unskilled	348 (74.5)
Homemaker/unemployed/retired	119 (25.5)
***Behavioral factors***	
Leisure-time physical activity, *n* (%)	
Inactive	375 (80.3)
Active	92 (19.7)
Fruits and vegetable servings^a^, *n* (%)	
≥5 servings per day	317 (68.5)
<5 servings per day	146 (31.5)
Alcohol consumption, *n* (%)	141 (30.2)
Tobacco use^b^, *n* (%)	118 (25.3)
***Anthropometric measures***	
BMI categories^c^, *n* (%)	
Normal weight (<23 kg/m^2^)	125 (26.8)
Overweight (≥23.0 and <25 kg/m^2^)	115 (24.7)
Obese (≥25 kg/m^2^)	226 (48.5)
Waist circumference in cm, mean (SD)	89.6 (9.6)
Fat percent (%), mean (SD)	29.1 (8.5)
***Psychosocial factors***	
Anxiety^d^, *n* (%)	
No	347 (74.8)
Yes	117 (25.2)
Depression^e^, *n* (%)	
No	314 (69.2)
Yes	140 (30.8)
Chronic stress^f^, *n* (%)	
None	158 (35.7)
Low	160 (36.3)
High	125 (28.2)
Self-perceived health status^f^, *n* (%)	
Good-excellent	163 (34.9)
Fair-poor	304 (65.1)
Social support score^g^, *n* (%)	23.6 (4.8)
***Clinical measures***	
Family history of CVD	116 (24.8)
Systolic BP in mmHg, mean (SD)	141.1 (19.5)
Diastolic BP in mmHg, mean (SD)	85.6 (12.5)
Fasting plasma glucose (mmol/L), mean (SD)	6.7 (2.3)
Two-hour plasma glucose (mmol/L), mean (SD)	7.9 (4.5)
Total cholesterol (mmol/l), mean (SD)	227.1 (42.7)
LDL cholesterol (mmol/l), mean (SD)	159.6 (36.9)
***Health utilization***	
Outpatient services in the last 4 weeks	132 (28.3)

a *One serving of fruit equals a medium-sized fruit or two small-sized fruits or half a glass of fruit juice or a bowl of grapes. One serving of vegetables (including tubers) equals 80 g.*

b *Tobacco use include smoke and smokeless (chewing tobacco and snuff) in the past 30 days.*

c *BMI was categorized according to the Indian guideline (32).*

d *Anxiety was measured using the General Anxiety Disorder scale (27).*

e *Depression was measured using Patient Health Questionnaire-9 amended in line with CURES-65 study (28).*

f *Self-perceived health status was measured using Short Form-36 (30).*

g *Social support was measured by the ENRICHED social support scale (31). It consists of 7 items with 5 scale (total score = 35), higher score indicates higher level of social support.*

*SD, standard deviation; BMI, body mass index; Percentages may not add up to 100% due to rounding*.

**Table 2:** Awareness, treatment, and control of hypertension among individuals with hypertension by sex at baseline (*n* = 467)^a^.

	**Total** **(*n* = 467)**	**Men** **(*n* = 308)**	**Women** **(*n* = 159)**	***P*-value^**b**^**
**Awareness**, ***n*** **(%)**	254 (54.4)	144 (46.8)	110 (69.2)	0.000
**Treatment**, ***n*** **(%)**				
Among those were aware	119 (46.9)	63 (43.8)	56 (50.9)	0.257
Among all cases	119 (25.5)	63 (20.5)	56 (35.2)	0.001
**Control**, ***n*** **(%)**				
Among those who were treated^c^	82 (68.9)	40 (63.5)	42 (75)	0.176
Among those with no treatment^d^	88 (25.3)	43 (17.6)	45 (43.7)	0.000
Among all cases	170 (36.4)	83 (26.9)	87 (54.7)	0.000

a *Data shown was among those with hypertension, defined as either systolic BP ≥ 140 mmHg or diastolic BP ≥ 90 mmHg or having anti-hypertension drugs, or self-reported having hypertension (n = 467).*

b *P-values are based on Chi square test between men and women.*

c *There were 119 participants were treated (63 men and 56 women).*

d *There were 348 participants were hypertensive but not receiving treatment (245 men and 103 women)*.

**Figure 2:**
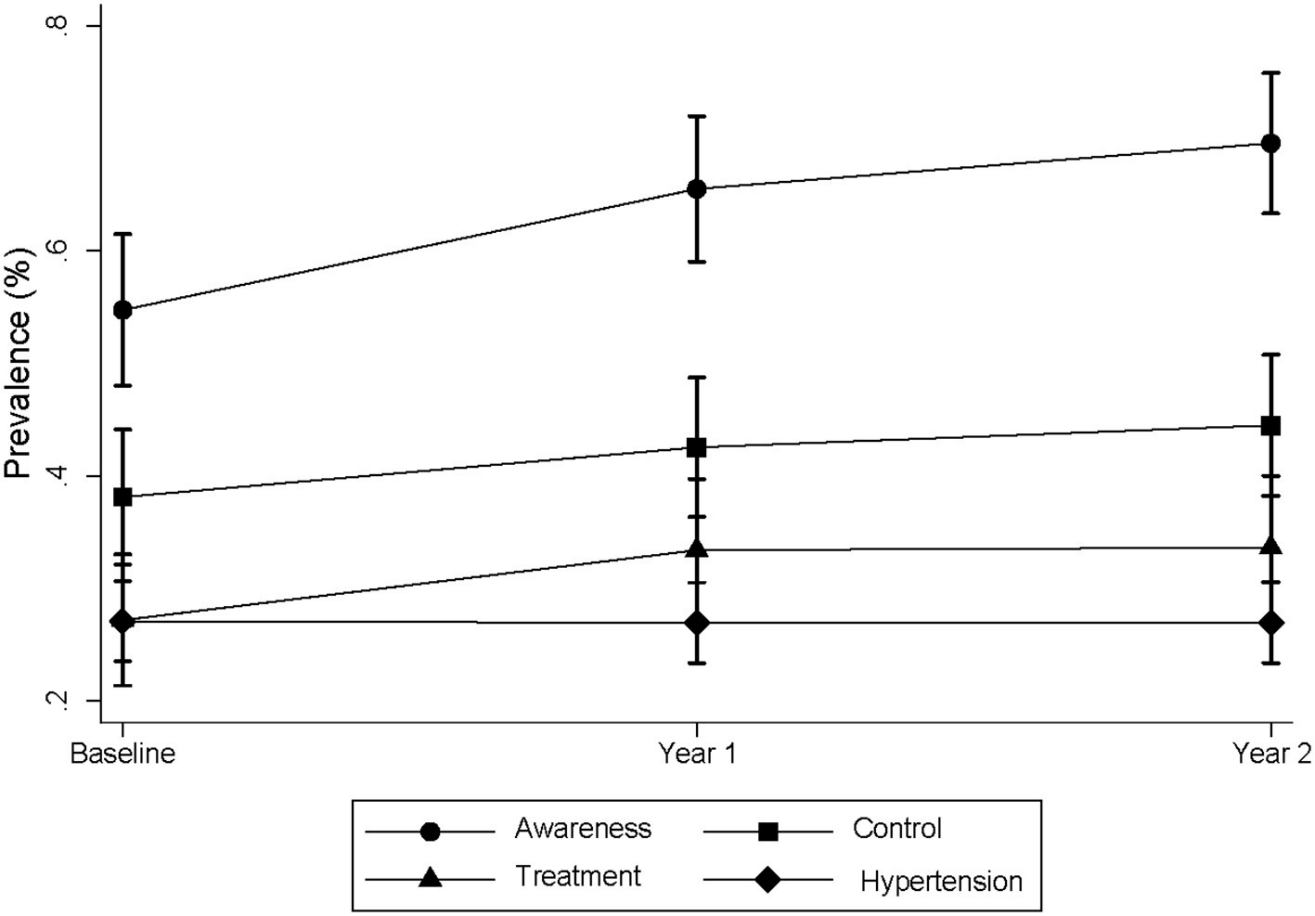
Trends of prevalence of hypertension awareness, treatment and control cross 2 years. The data was obtained from the K-DPP participants with repeated measures. There were 1,007 participants at baseline, 981 at year 1 and 962 at year 2. For hypertension, there were 274 at baseline, 265 at year 1 and 260 at year 2. The prevalence of awareness, treatment, and control were calculated among those who were hypertensive per definition at each time point.

There was a significant difference in hypertension awareness, treatment, and control by sex. On average, women had better hypertension management, compared to men. Even among those who had controlled hypertension but not on treatment, women were more than twice as likely to have their hypertension controlled, as compared to men (43.7 vs. 17.6%) (Table 2). The trends of these rates over 2 years based on repeated measures (*n* = 1,007) are presented in Figure 2. In brief, there was no change in the prevalence of hypertension over 2 years. However, there was a significant improvement in hypertension awareness (54% at baseline, 66% at year 1 and 70% at year 2, p for trend <0.001). Although no significant trends found for the other rates across 2 years, there was a significant increase in treatment and control rates from baseline to year 1 (27.4–34.4% and 38–42.6%, respectively). There was a modest increase of these rates from year 1 to year 2, without significant differences (Figure 2).

Baseline characteristics of hypertensive participants by hypertension awareness, treatment, and control are shown in Table 3. Men, those who did skilled/unskilled jobs, and who consumed alcohol were more likely to be unaware of their hypertension, and less likely to be receiving treatment or to have their BP controlled. On the other hand, those who with higher body fat, depressive symptoms, anxiety symptoms, chronic stress, poor to fair self-perceived health status and those who sought outpatient service in the last 4 weeks, were more likely to be aware and receive treatment and control their hypertension. Surprisingly, participants with higher social support were less likely to have their hypertension controlled as compared to those with lower level of social support.

**Table 3:** Characteristics of participants by hypertension awareness, treatment, and control groups among those with hypertension at baseline (*n* = 467)^a^.

	**Awareness** ^**b**^			**Treatment** ^**c**^			**Control** ^**d**^		
	**Yes** **(*n* = 254)**	**No** **(*n* = 213)**	***p*-value**	**Yes** **(*n* = 119)**	**No** **(*n* = 348)**	***p*-value**	**Yes** **(*n* = 170)**	**No** **(*n* = 297)**	***p*-value**
***Demographic factors***									
Age (years), mean (SD)	47.4 (8.2)	47.7 (7.9)	0.76	49.1 (8.0)	47.0 (8.0)	0.017	47.0 (8.2)	47.9 (7.9)	0.24
Men, *n* (%)	144 (56.7)	164 (77.0)	<0.001	63 (52.9)	245 (70.4)	<0.001	83 (48.8)	225 (75.8)	<0.001
Marital status, *n* (%)			0.88			0.53			0.93
Married	245 (96.5)	206 (96.7)		116 (97.5)	335 (96.3)		164 (96.5)	287 (96.6)	
Not married (separated/divorced/widowed/ never married)	9 (3.5)	7 (3.3)		3 (2.5)	13 (3.7)		6 (3.5)	10 (3.4)	
Education, *n* (%)			0.52			0.75			0.29
Up to primary school	76 (29.9)	66 (31.0)		33 (27.7)	109 (31.3)		54 (31.8)	88 (29.6)	
Secondary school	148 (58.3)	115 (54.0)		69 (58.0)	194 (55.7)		99 (58.2)	164 (55.2)	
Tertiary and above	30 (11.8)	32 (15.0)		17 (14.3)	45 (12.9)		17 (10.0)	45 (15.2)	
Occupation, *n* (%)			<0.001			<0.001			<0.001
Skilled/unskilled	170 (66.9)	178 (83.6)		70 (58.8)	278 (79.9)		109 (64.1)	239 (80.5)	
Homemaker/unemployed/retired	84 (33.1)	35 (16.4)		49 (41.2)	70 (20.1)		53 (31.2)	50 (16.8)	
***Behavioral factors***									
Leisure time physical active, *n* (%)	43 (16.9)	49 (23.0)	0.10	19 (16.0)	73 (21.0)	0.24	26 (15.3)	66 (22.2)	0.070
≥5 servings of vegetables and fruits/per day^e^, *n* (%)	73 (29.0)	73 (34.6)	0.19	32 (27.1)	114 (33.0)	0.23			
Alcohol consumption, *n* (%)	60 (23.6)	81 (38.0)	<0.001	22 (18.5)	119 (34.2)	0.001	30 (17.6)	111 (37.4)	<0.001
Current tobacco use^f^, *n* (%)	56 (22.0)	62 (29.1)	0.080	21 (17.6)	97 (27.9)	0.027	37 (21.8)	81 (27.3)	0.19
***Anthropometrics***									
Waist circumference (cm), mean (SD)	89.5 (10.3)	89.7 (8.7)	0.84	89.8 (10.3)	89.5 (9.3)	0.77	88.7 (10.6)	90.1 (8.9)	0.15
BMI categories^g^, *n* (%)			0.072			0.038			0.039
Normal weight (<23 kg/m^2^)	61 (24.1)	64 (30.0)		29 (24.4)	96 (27.7)		42 (24.7)	83 (28.0)	
Overweight (≥23.0 and <25 kg/m^2^)	57 (22.5)	58 (27.2)		21 (17.6)	94 (27.1)		33 (19.4)	82 (27.7)	
Obese (≥25 kg/m^2^)	135 (53.4)	91 (42.7)		69 (58.0)	157 (45.2)		95 (55.9)	131 (44.3)	
Body fat percent (%), mean (SD)	30.5 (8.9)	27.4 (7.8)	<0.001	31.4 (9.3)	28.3 (8.1)	<0.001	31.3 (9.0)	27.8 (8.0)	<0.001
***Psychosocial factors***									
Anxiety^h^, *n* (%)			<0.001			<0.001			<0.001
No	169 (67.1)	178 (84.0)		70 (59.3)	277 (80.1)		107 (63.7)	240 (81.1)	
Yes (mild-severe)	83 (32.9)	34 (16.0)		48 (40.7)	69 (19.9)		61 (36.3)	56 (18.9)	
Depression^i^, *n* (%)			<0.001			<0.001			
No	145 (58.7)	169 (81.6)		62 (54.9)	252 (73.9)		99 (60.7)	215 (73.9)	0.004
Yes (mild-severe)	102 (41.3)	38 (18.4)		51 (45.1)	89 (26.1)		64 (39.3)	76 (26.1)	
Chronic stress^j^, *n* (%)			0.004			0.017			0.10
None	76 (31.1)	82 (41.2)		35 (30.7)	123 (37.4)		49 (30.1)	109 (38.9)	
Low	84 (34.4)	76 (38.2)		35 (30.7)	125 (38.0)		60 (36.8)	100 (35.7)	
High	84 (34.4)	41 (20.6)		44 (38.6)	81 (24.6)		54 (33.1)	71 (25.4)	
Self-perceived health status^k^, *n* (%)			<0.001			0.15			0.022
Good-excellent	71 (28.0)	92 (43.2)		35 (29.4)	128 (36.8)		48 (28.2)	115 (38.7)	
Poor-fair	183 (72)	121 (56.8)		84 (70.6)	220 (63.2)		122 (71.8)	182 (61.3)	
Social support score^l^, mean (SD)	23.4 (4.9)	23.9 (4.7)	0.21	23.6 (4.5)	23.6 (4.9)	0.94	22.9 (5.0)	24.1 (4.6)	0.011
***Clinical measures***									
Family history of CVD, *n* (%)	72 (28.3)	44 (20.7)	0.055	32 (26.9)	84 (24.1)	0.55	49 (28.8)	67 (22.6)	0.13
Systolic BP (mmHg), mean (SD)	133.8 (21.0)	149.8 (13.0)	<0.001	134.4 (18.9)	143.3 (19.2)	<0.001	122.2 (10.9)	151.9 (14.5)	<0.001
Diastolic BP (mmHg), mean (SD)	81.8 (13.4)	90.2 (9.6)	<0.001	81.3 (12.3)	87.1 (12.2)	<0.001	74.9 (8.0)	91.8 (10.3)	<0.001
Fasting plasma glucose (mmol/L), mean (SD)	6.7 (2.2)	6.8 (2.3)	0.59	6.8 (2.4)	6.7 (2.2)	0.46	6.5 (1.8)	6.9 (2.5)	0.065
Two-hour plasma glucose (mmol/L), median (SD)	7.8 (4.3)	8.0 (4.6)	0.49	8.3 (4.7)	7.8 (4.4)	0.30	7.6 (4.0)	8.1 (4.7)	0.32
Total cholesterol (mg/dl), mean (SD)	224.3 (40.7)	230.5 (45.0)	0.15	220.8 (41.7)	229.2 (43.0)	0.095	219.7 (41.7)	231.5 (42.8)	0.008
LDL cholesterol (mg/dl), mean (SD)	158.1 (36.5)	161.4 (37.3)	0.38	155.4 (38.8)	161.0 (36.2)	0.20	154.6 (37.9)	162.6 (36.0)	0.040
***Health utilization***									
Outpatient service within last 4 wks, *n* (%)	102 (40.2)	30 (14.1)	<0.001	53 (44.5)	79 (22.7)	<0.001	75 (44.1)	57 (19.2)	<0.001

a *Hypertension was defined as mean systolic BP ≥ 140 mmHg or diastolic BP ≥ 90 mmHg based on the JNC 7 classification of hypertension, or currently under anti-hypertensive medication or self-reported hypertension.*

b *Awareness was defined as self-reported previous diagnosis of hypertension by a physician among those with hypertension (refer to #a).*

c *Treatment was defined as currently prescribed anti-hypertension medication.*

d *Controlled was defined as systolic BP <140 mmHg and diastolic BP <90 mmHg among those hypertensive participants (refer to #a).*

e *One serving of fruit equals a medium-sized fruit or two small-sized fruits or half a glass of fruit juice or a bowl of grapes. One serving of vegetables (including tubers) equals 80 g.*

f *Tobacco use include smoke and smokeless (chewing tobacco and snuff) in the past 30 days.*

g *BMI was categorized according to the Indian guideline (32).*

h *Anxiety was measured using the General Anxiety Disorder scale (27).*

i *Depression was measured using Patient Health Questionnaire-9 amended in line with CURES-65 study (28).*

j *Chronic stress was measured by Chronic stress scale used in MESA study (29).*

k *Self-perceived health status was measured using Short Form-36 (30).*

l *Social support was measured by the ENRICHED social support scale (31). It consists of 7 items with 5 scale (total score = 35), higher score indicates higher level of social support.*

*SD, standard deviation; BMI, body mass index; Percentages may not add up to 100% due to rounding*.

The cross-sectional results of correlates of hypertension awareness, treatment, and control are presented in Table 4. Participants with higher age (OR 0.96, 95% CI 0.94–0.99) and being men (OR 0.34, 95% CI 0.14–0.83) were less likely to have their hypertension controlled. Homemakers/unemployed/retired (OR 2.67, 95% CI 1.33–5.40) were more likely to be receiving hypertension treatment. Alcohol consumption (OR 0.49, 95% CI 0.27–0.87) were associated with uncontrolled hypertension. Psychosocial factors such as depression (OR 2.04, 95% CI 1.15–3.61) and fair to poor self-perceived health status (OR 1.87, 95% CI 1.15–3.04) were associated with increased hypertension awareness, whereas anxiety (OR 1.97, 95% CI 1.04–3.71) was associated with increased hypertension treatment. Social support (OR 0.95, 95% CI 0.90–1.00) was associated with uncontrolled hypertension. Seeking outpatient service within last 4 weeks was consistently associated with hypertension awareness (OR 3.78, 95% CI 2.19–6.53), treatment (OR 2.69, 95% CI 1.55–4.65), and control (OR 3.67, 95% CI 2.23–6.03).

**Table 4:** Factors associated with hypertension awareness, treatment, and control at baseline (cross-sectional analysis among hypertensive participants, *n* = 467)^a^.

	**Awareness**	**Treatment**	**Control**
	**OR (95% CI)**	**OR (95% CI)**	**OR (95% CI)**
***Demographic factors***			
Age (years)	0.99 (0.96, 1.02)	1.03 (0.10, 1.06)	**0.96 (0.94, 0.99)**
Men	0.77 (0.30, 1.93)	2.31 (0.85, 6.31)	**0.34 (0.14, 0.83)**
Occupation			
Skilled/unskilled	1.00	1.00	1.00
Homemaker/ unemployed/ retired	1.81 (0.88, 3.71)	**2.67 (1.33, 5.40)**	1.25 (0.67, 2.34)
***Behavioral factors***			
Alcohol consumption			
No	1.00	1.00	1.00
Yes	0.74 (0.44, 1.29)	0.57 (0.28, 1.14)	**0.49 (0.27, 0.87)**
Tobacco use			
No		1.00	1.00
Yes	–	0.66 (0.30, 1.46)	–
Body fat percent	1.01 (0.97, 1.06)	1.03 (0.98, 1.08)	0.98 (0.94, 1.02)
***Psychosocial factors***			
Anxiety			
No	1.00	1.00	1.00
Yes (mild-severe)	1.08 (0.58, 2.03)	**1.97 (1.04, 3.71)**	1.63 (0.93, 2.86)
Depression			
No	1.00	1.00	1.00
Yes (mild-severe)	**2.04 (1.15, 3.61)**	1.30 (0.72, 2.35)	0.92 (0.54, 1.56)
Chronic stress			
None	1.00	1.00	1.00
Low	0.99 (0.59, 1.68)	0.69 (0.38, 1.28)	–
High	1.10 (0.59, 2.05)	0.93 (0.47, 1.82)	–
Self-perceived health status			
Good-excellent	1.00	1.00	1.00
Fair-poor	**1.87 (1.15, 3.04)**	–	1.25 (0.77, 2.01)
Social support score	–	–	**0.95 (0.90, 1.00)**
***Heath utilization***			
Outpatient service within last 4 weeks			
No	1.00	1.00	1.00
Yes	**3.78 (2.19, 6.53)**	**2.69 (1.55, 4.65)**	**3.67 (2.23, 6.03)**

a *Results obtained from multi-level mixed logistic regression model, considering (cluster as level 2) among hypertensive participants at baseline.*

*Bold values indicate statistically significant at p < 0.05.*

*Variables selected in each model was based on the results of univariate analyses, some variables were included in one model but not in others, therefore represented as “–.”*

Longitudinal associations between these factors and hypertension awareness, treatment, and control revealed similar results (Table 5), including (1) being homemakers/unemployed/retired (OR 2.09, 95% CI 1.25–3.47) were more likely to receive treatment; (2) those who consumed alcohol (OR 0.49, 95% CI 0.31–0.80) was associated with uncontrolled hypertension; (3) fair to poor self-perceived health status (OR 1.87, 95% CI 1.15–3.04) were associated with increased hypertension awareness; (4) seeking outpatient service within last for weeks was consistently associated with better hypertension awareness (OR 1.90, 95% CI 1.27–2.87), treatment (OR 1.73, 95% CI 1.20–2.50) and control (OR 1.96, 95% CI 1.37–2.80). Differently, body fat percent was associated with slightly increased hypertension treatment (OR 1.04, 95% CI 1.00–1.08) and lower hypertension control (OR 0.97, 95% CI 0.94–1.00). No association was found between social support and hypertension control.

**Table 5:** Factors associated with hypertension awareness, treatment, and control over 2-year follow-up among hypertensive participants^a^.

	**Awareness**	**Treatment**	**Control**
	**OR (95% CI)**	**OR (95% CI)**	**OR (95% CI)**
***Demographic factors***			
Age (years)	**0.97 (0.95, 1.00)**	**1.04 (1.01, 1.06)**	0.98 (0.96, 1.00)
Men	0.70 (0.33, 1.47)	1.77 (0.86, 3.63)	**0.27 (0.14, 0.53)**
Occupation			
Skilled/unskilled	1.00	1.00	1.00
Homemaker/ unemployed/ retired	1.72 (0.98, 3.02)	**2.08 (1.25, 3.47)**	1.28 (0.80, 2.04)
***Behavioral factors***			
Alcohol consumption			
No	1.00	1.00	1.00
Yes	0.82 (0.51, 1.29)	0.91 (0.54, 1.52)	**0.49 (0.31, 0.80)**
Tobacco use			
No		1.00	1.00
Yes	–	0.70 (0.37, 1.35)	–
Body fat percent	1.01 (0.97, 1.05)	**1.04 (1.00, 1.08)**	**0.97 (0.94, 1.00)**
***Psychosocial factors***			
Anxiety			
No	1.00	1.00	1.00
Yes (mild-severe)	1.20 (0.70, 2.07)	1.47 (0.89, 2.24)	0.99 (0.62, 1.58)
Depression			
No	1.00	1.00	1.00
Yes (mild-severe)	1.48 (0.91, 2.42)	1.17 (0.74, 1.86)	0.90 (0.58, 1.38)
Chronic stress			
None	1.00	1.00	1.00
Low	0.76 (0.50, 1.15)	0.72 (0.48, 1.09)	–
High	0.96 (0.57, 1.62)	1.05 (0.65, 1.70)	–
Self-perceived health status			
Good-excellent	1.00	1.00	1.00
Fair-poor	**1.80 (1.23, 2.62)**	–	1.37 (0.96, 1.94)
Social support score	**–**	**–**	1.01 (0.97, 1.04)
***Heath utilization***			
Outpatient service within last 4 weeks			
No	1.00	1.00	1.00
Yes	**1.90 (1.27, 2.87)**	**1.73 (1.20, 2.50)**	**1.96 (1.37, 2.80)**

a *Results obtained from multi-level mixed logistic regression model, considering (cluster as level 2) in K-DPP trial participants among hypertensive participants over 2-year period. With missing observations at each time point, the number of participants with hypertension were 274 at baseline, 265 at year one and 260 at year two, respectively.*

*Bold values indicate statistically significant at p < 0.05.*

*Variables selected in each model was based on the results of univariate analyses, some variables were included in one model but not in others, therefore represented as “–.”*

## Discussion

To our knowledge, this is the first study in India to investigate a comprehensive range of factors in relation to hypertension awareness, treatment, and control, both cross-sectionally and longitudinally. The study has identified the importance of targeting males and alcohol consumption in relation hypertension management, as well as the importance of psychosocial factors and health utilization. Although more than half of the participants in this study with hypertension were aware of their condition, only a quarter of them were receiving treatment. Despite receiving treatment, <70% participants had their hypertension controlled. A quarter of those who were not receiving treatment had their hypertension controlled, leading to more than 50% of those who were controlled of their hypertension was not dependent on treatment (*n* = 88). Addressing key risk behaviors and psychosocial factors as well as health utilization may help better manage hypertension and guide policies and promotion strategies in a country like India in the future.

Compared with previous systematic reviews on the region-specific prevalence of hypertension which found about 25% of rural population and 21% of the South India population had hypertension (33), about 27% of participants in our study (mostly rural residents) were found to have hypertension. The level of hypertension awareness, treatment, and control in previous studies conducted in Kerala varies across districts and age groups, with lower awareness found in Trivandrum (16.8%) (34), Kannur district (38.7%) (14), and higher in Ernakulam district with older age groups (78%) (15), comparing to 54% found in our study in rural Kerala. Compared with one of the earliest cohorts in Kerala to study the incidence of hypertension (35), we found slightly higher rates of awareness (54.4 vs. 42.9%) and treatment (25.5 vs. 22.9%). Interestingly, all these studies found higher treatment rates than control rates among all hypertensive participants; however, we found a lower rate of treatment (25.5%), than control (36.4%). The reason could be that in our study, more than 50% of controlled hypertension were not using any anti-hypertensive treatment, leading to a possible higher rate of control than treatment. This is an important finding, because most studies that reported controlling hypertension, have been among those with treatment. However, ignoring those who have been able to control their hypertension without treatment may underestimate the importance of non-pharmaceutical management in hypertension, including lifestyle modification and psychosocial factors (36).

Men were found to have poorer hypertension control, compared to women. This is consistent with the results from 44 LMICs that men are less likely to reach each step of hypertension care cascade, (37) as well as other studies (8, 38). The possible reasons for this difference might be due to sex norms and maternal health focused services in LMICs. Interestingly, one study in Nepal found although women were more likely to be aware and treated for hypertension, they had lower control rates compared to men, which might be partly due to inequality issues in hypertension management (39). In our study, even among those who were not on treatment, but had their hypertension controlled, women were twice likely to control their hypertension, compared to men. This indicates that women may manage non-pharmaceutical related factors (e.g., lifestyle factors) better than men or that there are some other important sex specific factors.

Alcohol consumption was found to be associated with uncontrolled hypertension in both cross-sectional and longitudinal analyses. Different physiological mechanisms of alcohol in raising BP have been demonstrated in the literature previously (40). Meta-analysis of intervention trials have confirmed a dose-dependent manner between alcohol reduction and blood pressure reduction (41). The findings of the meta-analysis indicated the necessity of prioritizing reducing alcohol consumption in countries with substantial alcohol-attributable risk in hypertension management and health promotion. In addition, sex-specific alcohol consumption and hypertension incidence has been suggested in a systematic review of high quality cohorts (42), that found a dose relationship between alcohol consumption (any level) and incidence of hypertension in men but not women. In our study, alcohol consumption was very uncommon for women, which has identified the importance of addressing men related risk behaviors in hypertension management in countries like India.

Besides alcohol consumption, body fat percent was found to be positively associated with hypertension treatment, but uncontrolled hypertension in the longitudinal analysis. This indicates that although patients with a higher fat distribution are likely to get treatment, the treatment doesn't necessarily lead to an optimal control of hypertension. This is different from the other study, where body fact percent was found to be positively associated across all hypertension outcomes, including controlled hypertension (9). However, the authors did not discuss the potential mechanisms, and given the cross-sectional study design, the results need to be confirmed in prospective studies. In fact, there are different pathophysiological mechanisms have been established in obesity induced hypertension (43). Maintaining a healthy body weight and reasonable body fat percent are important in hypertension control.

Anxiety and depressive symptoms were found to be strongly associated with higher level of hypertension awareness and treatment, respectively, although only from the cross-sectional analysis. Previous studies have also found a higher level of psychological distress in participants who were aware of their hypertension (44). It might be that those who were more distressed were more likely to worry about their health, so they were more likely to seek health advice. However, we did not find this association in the longitudinal analysis. Unexpectedly, we also found anxiety was associated with a higher chance of getting hypertension treated, which was inconsistent with previous studies that found anxiety was associated with non-adherence to hypertension treatment (45). However, such association supports the finding that people with anxiety were more likely to be aware of their hypertension, because they were more likely to get hypertension treated once they were aware of their hypertension. Alternatively, this might also be an inverse association due to the cross-sectional nature that people who got treated were more likely to get anxious due to unsatisfactory outcomes. Nevertheless, as about 25–30% of hypertensive participants had anxiety or depression in our study, addressing mental health in hypertension management may further help to understand each step of the cascade care of hypertension particularly in settings like India, where mental health has been suggested to be integrated in cardiovascular diseases management (46).

Similar association was found between poor to fair self-perceived health status and a higher awareness of hypertension. It is known that people with hypertension were more likely to have lower satisfaction of their health status than normotensive counterparts (13). So such association could also be an inverse association observed in the cross-sectional manner. However, the same direction was confirmed in the longitudinal analysis as well, which may indicate that the potential role of self-perceived health in early hypertension awareness and diagnosis. Longer follow-up studies are needed to confirm such association.

Although higher social support was associated with uncontrolled hypertension, this was not supported by the findings from longitudinal analysis. Social factors have been suggested to be associated with better hypertension diagnosis and control (47). Interestingly, in an Albanian study that included multi-level of social and community determinants found that children's support was associated with uncontrolled hypertension, but support from friends was associated with controlled hypertension (10). The authors concluded that this may be partly due to traditional “familism” and children's support, which may present unwanted responsibility and potential conflicts. Nevertheless, the inverse association between social support and hypertension control may also be due to inverse causation, that people who with uncontrolled hypertension were getting more social support. In fact, social support seemed to be associated with controlled hypertension from longitudinal analysis, despite non-significant result.

Another finding from this study was that seeking outpatient service was consistently associated with hypertension awareness, treatment, and control from both cross-sectional and longitudinal analyses. Frequently seeking health service was reported to be associated with better awareness and treatment of hypertension in another India study (9). It is likely that frequent outpatient service did play a role in improving hypertension awareness, treatment, and control. This again provides some evidence of the importance of providing access to outpatient services in rural settings to improve hypertension management.

One of the key strengths of this study is that we were able to conduct longitudinal analysis of the participants from K-DPP trial, which enabled us to identify potential predictors of hypertension awareness, treatment, and control. Another strength is that we have included a comprehensive range of factors, including more traditional demographic and behavioral factors, as well as psychosocial factors and health utilization variables. Despite these strengths, there were some limitations. Firstly, longitudinal study results are from K-DPP trial participants, who had been identified as having an elevated risk of diabetes. However, those participants had been randomly selected from the community and their socio demography (age, education, occupation, marital status and household size) were very similar to the general population of rural Kerala (48). Secondly, we did not have information on community and societal level factors such as quality of health care and perception of safety. Lastly, we do not have information on the duration of hypertension, but we were able to detect newly diagnosed hypertension in this study.

In conclusion, our findings highlight the potentially important role of psychosocial factors and health service utilization in hypertension management. It is probably also important to give more attention to alcohol consumption in Indian males as well as body fat control. These factors need to be considered in future health promotion strategies directed at the prevention and control of hypertension in India. Furthermore, more longitudinal studies in the general population and studies in other states of India on the determinants of hypertension management are needed, as the current findings in Kerala might be a harbinger of the situation in the rest of India over the coming years. In addition, our findings need to be interpreted properly, considering Indian specific attributes such as the high prevalence of hypertension patients self-medication, healthcare policy and financial burden in patients, as well as quality of health care. These can be further studied in future studies to facilitate policy makings related to hypertension management in India.

## Data Availability Statement

The original contributions presented in the study are included in the article/supplementary material, further inquiries can be directed to the corresponding author.

## Ethics Statement

The studies involving human participants were reviewed and approved by the SreeChitra Tirunal Institute for Medical Sciences and Technology, Trivandrum in India (SCT/IEC-333/May 2011), Monash University (CF11/0457-2011000194), and the University of Melbourne in Australia (1441736). The patients/participants provided their written informed consent to participate in this study.

## Author Contributions

YC and TS contributed to conceptualization of the study. YC carried out the data analyses and wrote the manuscript. TS contributed to critically reviewing the analysis. TS, TH, NK, GM, YW, and BO contributed to critically review of the manuscript and approved for the final version of the study. All authors contributed to the article and approved the submitted version.

## Funding

This research was supported by funding from the National Health and Medical Research Council, Australia (1005324) and (1160283) for the original and follow-up study of the K-DPP study respectively.

## Conflict of Interest

The authors declare that the research was conducted in the absence of any commercial or financial relationships that could be construed as a potential conflict of interest.

## Publisher's Note

All claims expressed in this article are solely those of the authors and do not necessarily represent those of their affiliated organizations, or those of the publisher, the editors and the reviewers. Any product that may be evaluated in this article, or claim that may be made by its manufacturer, is not guaranteed or endorsed by the publisher.
